# Accuracy of full arch scans performed with nine different scanning patterns– an in vitro study

**DOI:** 10.1007/s00784-025-06154-2

**Published:** 2025-01-27

**Authors:** Kerstin Schlögl, Jan-Frederik Güth, Tobias Graf, Christine Keul

**Affiliations:** 1 Department of Prosthetic Dentistry, LMU University Hospital, LMU Munich, Goethestrasse 70, 80336 Munich, Germany; 2 Department of Prosthetic Dentistry, Center for Dentistry and Oral Medicine (Carolinum), Goethe University Frankfurt am Main, Frankfurt am Main, Germany

**Keywords:** Full-arch digitization, Scanning strategies, Accuracy, Intraoral scanner

## Abstract

**Objective:**

Evaluation of the accuracy of direct digitization of maxillary scans depending on the scanning strategy.

**Materials and methods:**

A maxillary model with a metal bar as a reference structure fixed between the second molars was digitized using the CEREC Primescan AC scanner (*N* = 225 scans). Nine scanning strategies were selected (*n* = 25 scans per strategy), differing in scan area segmentation (F = full jaw, H = half jaw, S = sextant) and scan movement pattern (L = linear, Z = zig-zag, C = combined). Trueness was assessed by evaluating linear differences in the X, Y, and Z axes and angular deviations (α axial, α coronal, α total) compared to a reference dataset. Statistical differences were analyzed using Kruskal-Wallis and Mann-Whitney U tests (p<0.017). Precision was analyzed by the standard deviation of linear and angular aberrations (ISO 5725-1) (*p* < 0.05).

**Results:**

Strategy F_L_ showed significantly higher trueness and precision than F_Z_ for VE (*p* = 0.009), V_E_(y) (*p* = 0.010), α_overall_ (*p* = 0.004), and α_axial_ (*p* = 0.002). Strategy F_C_ demonstrated significantly better trueness than F_Z_ for VE (*p* = 0.007), α_overall_ (*p* = 0.010), and α_coronal_ (*p* = 0.013). For scan segmentation, F_L_ showed better trueness for V_E_(y) (*p* = 0.001) and α_axial_ (*p* < 0.001) than H_L_. Strategy H_L_ showed better trueness for V_E_(z) than for F_L_ and S_L_ (*p* = 0.001, *p* = 0.002). The scanning patterns F_L_, F_C_, and H_L_ exhibited the best performance for trueness and precision.

**Conclusions:**

Scanning motion and segmentation have a significant impact on the trueness and precision of full-arch scans.

**Clinical relevance:**

The scanning strategy is decisive in enhancing the clinical workflow and the accuracy of full-arch scans.

## Introduction

The digital workflow in dentistry has recently been transformed by the influence of computer-aided design (CAD) and computer-aided manufacturing (CAM) across nearly all clinical applications, such as orthodontics and prosthodontics. Modern digital technologies in dentistry now provide enhanced efficiency and superior quality in diagnostics, clinical monitoring, therapy planning, and restoration fabrication. Additionally, the standardization achieved through digital workflows reduces inaccuracies associated with conventional impression-taking and model fabrication [1]. Furthermore, documentation through direct digitization facilitates [1]more objective treatment decisions by relying on acquired diagnostic data [2, 3].

A complete digital workflow without the process of a conventional impression involves three coordinated steps: data acquisition, data processing, and restoration fabrication [4]. The accuracy of the scan, and consequently the data acquisition process, plays a decisive role in this initial step of the digital workflow, as the subsequent steps depend on the quality of this data. Several factors can influence this accuracy. In this context the scanning system and its calibration [5], experience of the operator [6] and the scanning strategy [7, 8] are critical determinants of the accuracy, in detail the trueness and precision of the resulting model data.

For evaluating the accuracy of full-jaw datasets, there is currently no standardized guideline available. Up to date, two methods are described in the literature. The first involves using a best-fit algorithm to superimpose test and reference data, allowing the calculation of metric deviations between the two datasets [9–11]. However, this method is limited by potential unrecognized misalignments introduced by the software algorithm during the alignment process. The second method uses metrical analysis of real geometric values obtained from reference objects, which are either fixed to an in vitro analysis model or attached to the patient’s arch in vivo [12–16].

The current consensus in literature is that the new generation of intraoral scanners demonstrate convincing accuracy for scans up to a quadrant, with equivalent or even superior accuracy of the generated virtual dental model [9]. However, scanning an entire jaw remains challenging [15, 17] as increased scan distances are associated with cumulative scanning and merging errors, resulting in higher inaccuracies, particularly for full-arch scans [18]. Notably, these inaccuracies depend on the specific systems utilized [19, 20].

Considering this, the question arises how potential sources of error in the scanning strategy affect the trueness and precision of digital data acquisition. Several studies indicate that accuracy improves with a more complex, non-linear scanning strategy [8, 21, 22], while others recommend adhering to the use of manufacturer’s suggested strategy [23]. Additionally, capturing undercuts requires rotation of the handpiece to enhance detection [15].

The aim of the present study is to systematically investigate the influence of different movement patterns and targeted scan segmentation on scan accuracy. This study evaluates trueness and precision in the in-vitro digitization of a maxillary model using a new generation IOS scanner (CEREC Primescan AC). The null hypothesis is that varying the scanning pattern will not result in significant differences intrueness or precision.

## Materials and methods

A maxillary full-arch model made of polyurethane (AlphaDie MF, LOT 2012008441; Schütz Dental GmbH, Rosbach, Germany) with a homogeneous, matt surface was used as analysis model to conduct the study. A metal bar was inserted in the area of the second molars and used as a reference structure (GARANT, DIN 875-00-g; Hoffmann Group, Munich, Germany).

### Acquisition of the reference dataset of the bar

The reference measurement of the metal bar was carried out with a coordinate measuring machine (CMM: Mitutoyo Crysta Apex C 574; Createch Medical Mendaro, Spain; software: MCOSMOS Mitutoyo Software; Mitutoyo, Neuss, Germany) before it was fixed on the analysis model. This measurement was performed at a temperature of 20 °C with a maximum permissible error (MPEe) of the CMM of 1.9 μm + (3*L/1000), where the parameter L is defines by the real length of the used metal bar. Subsequently, the STL dataset generated by the CMM was imported into the analysis software (Geomagic Control 2015; version: 2015.3.1.0, 64-bit, Geomagic, Morrisville, MC, US). The calculated reference length of the metal bar was 55.066 mm.

### Scanning of the upper jaw model

**Fig. 1:**
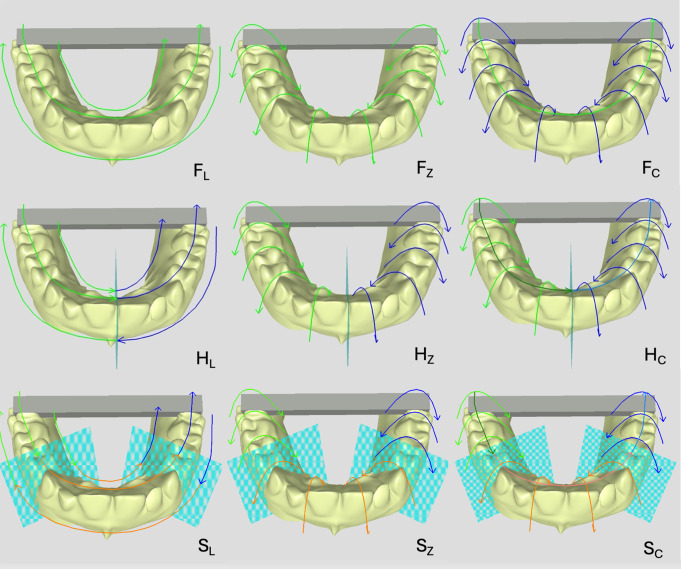
Visualization of the scanning strategies

CEREC Primescan AC (software version 2015.3.1.0, Dentsply Sirona, Bensheim, Germany) was used for all scans (*n* = 25/strategy). Nine different scanning strategies were developed for direct digitization, combining the segmentation of the scan area (F = full jaw, H = half jaw, and S = Sextant) and three different scan movement patterns (_L_ = linear, _Z_ = zig-zag, and _C_ = combined) as shown in Fig. 1. During the scan, it was ensured that a maximum of 20 mm of the bar ends were captured by the scanner to avoid connection of the complete bar in the virtual dataset. The full length of the bar was not scanned, due to the following reason: The reference bar contains no geometric structures for optimal merging the single captures of CEREC Primescan AC. Hence, the digitization of the complete bar was not possible without causing distortions in the complete arch scan, as the software algorithm of CEREC Primescan AC tried to connect both bar ends if the scanning area was too large.

An experienced operator [K.S.] performed all scans using the extraoral data acquisition mode of CEREC Primescan AC. Each scan was obtained under the same conditions with constant ambient light settings. At the beginning of each scan, the CEREC Primescan AC scanning device was calibrated using the “Calibration Set Primescan” according to manufacturer’s guidelines. A maximum of two scans were performed successively with a following break of 30 min, so that any influence by heating of the scanning device could be excluded.

### Data analysis and calculation of the parameters (linear parameters/angular parameters)

**Fig. 2:**
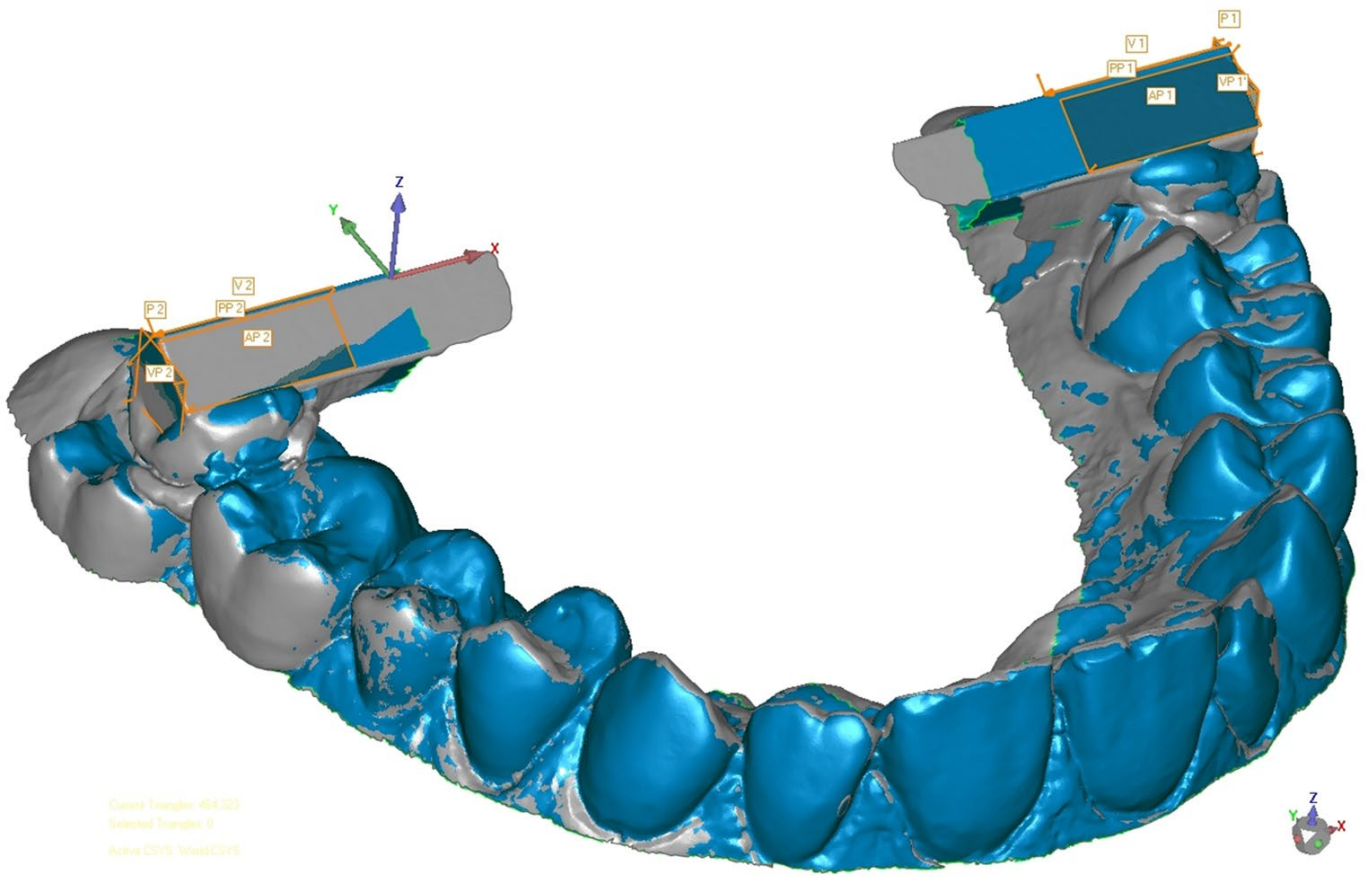
Construction of the surfaces in contact feature mode

Each scan (*N* = 225, *n* = 25 per group) was exported as an STL dataset from the respective scan software of CEREC Primescan AC and imported into the analysis software (Geomagic Control 2015). The data was virtually adjusted in a three-dimensional coordinate system, that included XZ-, XY-, and YZ-axes as the coronal, transversal and sagittal planes (Fig. 2). Using the “Contact Feature” mode of the analysis software, anterior surfaces (AP 1 and AP 2), posterior surfaces (PP 1 and PP 2), and vestibular surfaces (VP 1 and VP 2) were constructed at each bar end (Fig. 2).

**Fig. 3:**
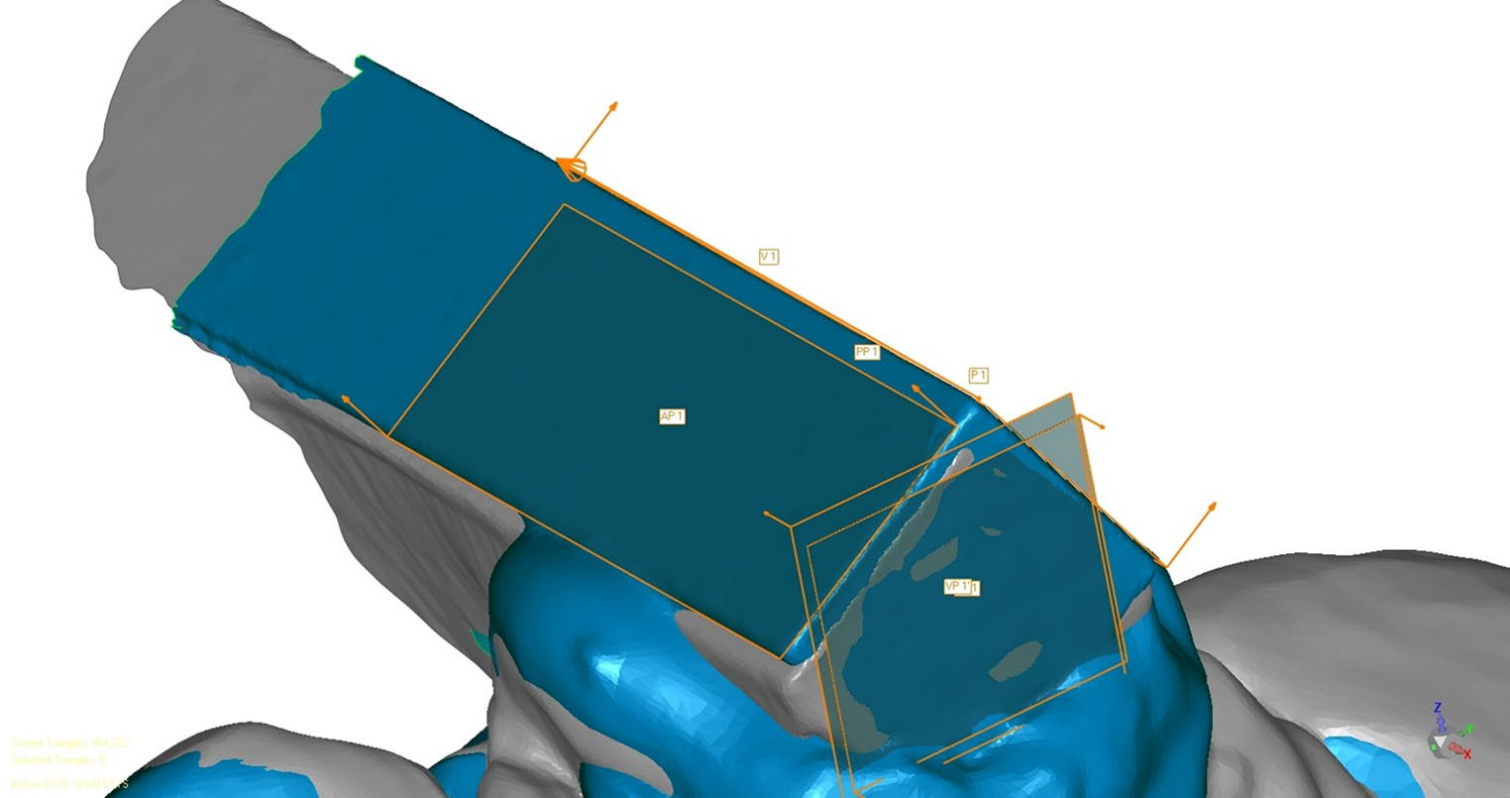
Construction of the surfaces in the first quadrant

By the intersection of the planes further vectors and points were defined: the intersection lines of the anterior and posterior surfaces resulted in the horizontal vectors $\:\stackrel{⃑}{V}$1 (AP1 and PP1) and $\vec{V}$2 (AP2 and PP2). The points P1 and P2 were determined as the intersections of $\vec{V}$1 and VP1 or $\vec{V}$2 and VP2, respectively (Fig. 3). For the metric analysis of the torsion in all three dimensions, the vestibular surface of the second quadrant (VP2) was parallel shifted by the calculated reference length of the metal bar (L = 55.066 mm) in the direction of the first quadrant to construct the surface VP2’. The surface VP2’ with the vector $\vec{V}$2 resulted in the constructed point P2’. To calculate the metric values of the torsion in the X-, Y- and Z- axes, the vectorial error $\vec{V}_E$ between P2’ and P1 was then analyzed using the calculation formula below (x, y, and z are the coordinates of the X-, Y-, and Z-axes):

$$\vec{V}_E=\left(\begin{array}{ll}x_{p 1}- & x_{p 2} \\y_{p 1}- & y_{p 2} \\z_{p 1}- & z_{p 2}\end{array}\right)$$

To determine the angular deviations of the upper bar edges, α_overall_ was first calculated as follows:

$$\eqalign{& {\alpha _{{\rm{overall }}}} = \cr & \alpha \cos {{{X_{V1}}*{X_{V2}} + {Y_{V1}}*{Y_{V2}} + {Z_{V1}}*{Z_{V2}}} \over {\sqrt {X_{V1}^2 + Y_{V1}^2 + Z_{V1}^2} *\sqrt {X_{V2}^2 + Y_{V2}^2 + Z_{V2}^2} }} \cr & *{{180} \over \pi } \cr} $$

In addition, the differentiated projection of α_overall_ on the coronal and horizontal planes gives further information about the direction of the angular torsion of the bar. It was calculated using the following equations (x, y, and z are the coordinates of the X-, Y-, and Z-axes):

$$\:\alpha\:\text{c}\text{o}\text{r}\text{o}\text{n}\text{a}\text{l}\:=\:\alpha\:\text{c}\text{o}\text{s}\:\:\:\frac{{X}_{V1}*{X}_{V2}+{Y}_{V1}*{Y}_{V2}}{\sqrt{{{X}_{V1}}^{2}+{{Y}_{V1}}^{2}}\:*\:\sqrt{{{X}_{V2}}^{2}+{{Y}_{V2}}^{2}}}\:*\:\frac{180}{\pi\:}$$

$$\:\alpha\:\text{a}\text{x}\text{i}\text{a}\text{l}\:=\:\alpha\:\text{c}\text{o}\text{s}\:\:\frac{{X}_{V1}*{X}_{V2}+{Z}_{V1}*{Z}_{V2}}{\sqrt{{{X}_{V1}}^{2}+{{Z}_{V1}}^{2}}\:*\:\sqrt{{{X}_{V2}}^{2}+{{Z}_{V2}}^{2}}}\:*\:\frac{180}{\pi\:}$$

### Statistical analysis

Statistical data analysis was performed using SPSS statistics software (version 26.0.0.0, IBM, Armonk, NY, USA). The significance level was set at 5% (*p* < 0.05). The Kolmogorov Smirnov and Shapiro-Wilk test was performed to assess the normal distribution.

To determine differences in the trueness between the scanning strategies (segmentation and movement), Kruskal-Wallis and the Mann-Whitney-U test with Bonferroni correction (*p* < 0.017) were used. For the analysis of precision (according to ISO 5725-1) the standard deviation was used [13].

## Results

**Fig. 4:**
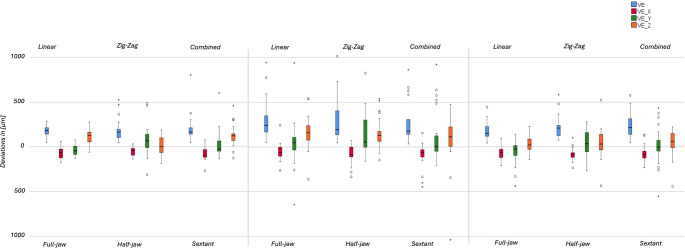
Statistical analysis of linear deviations

**Fig. 5:**
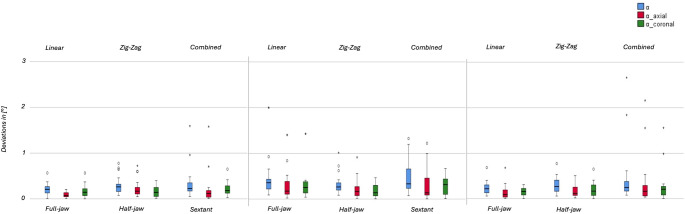
Statistical analysis of angular deviations

The deviations are expressed as median, minimum, maximum and standard deviation in Tables 1 and 2 including the 95% confidence interval for each parameter. The boxplots of all tested strategies are displayed in Figs. 4 and 5.

**Table 1:** Descriptive statistics of linear deviations with mean values (M), standard deviation (SD), median (MD) and 95% confidence interval (CI) of CEREC Primescan AC; uppercase superscript letters indicate significant differences between the scanning strategies regarding the trueness; lowercase superscript letters indicate significate differences between the scanning strategies regarding the precision

		ΔL (µm)	VE (µm)	VE(x) (µm)	VE(y) (µm)	VE(z) (µm)
Strategy FL	M	-64.94	171.53	-67.92	-34.46	121.22
	SD	58.84	59.41	59.03	60.58	75.32
	MIN	-169.07	49.77	-173.24	-126.79	(-59.77)
	**MED**	**-61.67** ^**A, a**^	**181.65** ^**A, C, a**^	**-65.13** ^**A, a**^	**-44.12** ^**A, a**^	**127.89** ^**A, a**^
	MAX	60.95	288.59	60.24	78.16	272.12
	95% CI	-89.24/-40.65	147.00/196.05	-92.28/-43.56	-59.46/-9.45	90.13/152.32
Strategy FZ	M	-66.59	301.86*	-50.30	41.59*	148.29
	SD	92.73	215.91	95.53	256.96	184.96
	MIN	-261.60	52.45	-262.30	-642.98	-360.63
	**MED**	**-59.09** ^**A, a**^	**242.12** ^**B, a**^	**-61.57** ^**A, a**^	**47.96** ^**B, a**^	**159.20** ^**A, B, a**^
	MAX	152.92	945.63	243.22	940.55	539.82
	95% CI	-104.87/-28.31	212.74/390.99	-89.73/-10.86	-64.47/147.66	71.94/224.64
Strategy FC	M	-63.45	175.91*	-61.31	-53.01*	26.16
	SD	73.98	88.16	80.35	132.94	91.23
	MIN	-208.70	43.61	-209.81	-439.21	-141.91
	**MED**	**-65.38** ^**A, a**^	**151.6** ^**C, A, a**^	**-65.42** ^**A, a**^	**-26.40** ^**A, B, a**^	**21.67** ^**B, a**^
	MAX	97.88	443.0	96.66	137.00	225.42
	95% CI	-93.99/-32.90	139.52/212.31	-94.48/-28.14	-107.89/1.86	-11.51/63.82
Strategy HL	M	-49.94	182.13*	-50.10	68.91*	12.97
	SD	46.27	116.81	46.27	163.53	107.61
	MIN	-134.28	44.50	-135.1	-313.18	-185.56
	**MED**	**-41.74** ^**A, a**^	**164.63** ^**A, a**^	**-42.44** ^**A, a**^	**68.7** ^**A, a**^	**2.77** ^**A, a**^
	MAX	33.76	524.96	33.41	484.95	186.92
	95% CI	-69.04/-30.82	133.91/230.36	-69.20/-31.00	1.42/136.41	-31.45/57.39
Strategy HZ	M	-79.20	303.54*	-81.18*	147.89*	153.65
	SD	96.24	237.87	99.16	241.0	174.42
	MIN	-304.23	45.93	-334.15	-157.98	-145.63
	**MED**	**-84.30** ^**A, a**^	**192.89** ^**A, a**^	**-86.04** ^**A, a**^	**56.16** ^**A, a**^	**130.41** ^**B, a**^
	MAX	70.98	1016.27	71.23	827.17	541.89
	95% CI	-118.94/-39.46	205.35/401.74	-122.11/-40.24	48.42/247.36	81.65/225.64
Strategy HC	M	-85.08	216.37*	-83.54	41.15	45.81*
	SD	61.04	120.51	67.58	141.54	165.74
	MIN	-236.11	75.88	-235.46	-263.70	-436.34
	**MED**	**-84.32** ^**A, a**^	**210.34** ^**A, a**^	**-85.03** ^**A, a**^	**34.34** ^**A, a**^	**31.84** ^**A, B, a**^
	MAX	16.72	584.13	102.13	279.91	522.57
	95% CI	-110.28/-59.87	166.62/266.12	-111.44/-55.64	-17.28/99.57	-22.61/114.23
Strategy SL	M	-76.23	205.54*	-74.59	19.34*	127.60
	SD	67.11	143.63	73.25	149.18	117.78
	MIN	-258.54	44.94	-266.69	-133.18	-125.56
	**MED**	**-92.44** ^**A, a**^	**164.93** ^**A, a**^	**-93.13** ^**A, a**^	**-24.96** ^**A, a**^	**123.22** ^**A, a**^
	MAX	74.67	803.07	78.61	601.37	460.59
	95% CI	-103.94/-48.53	146.25/264.83	-104.83/-44.35	-42.24/80.92	78.98/176.22
Strategy SZ	M	-86.05*	322.01*	-89.07*	115.89*	77.71*
	SD	132.44	313.47	132.91	283.71	282.10
	MIN	-448.36	33.68	-446.19	-204.45	-1037.96
	**MED**	**-60.13** ^**A, a**^	**177.74** ^**A, a**^	**-62.45** ^**A, a**^	**6.53** ^**A, a**^	**112.88** ^**A, a**^
	MAX	155.07	1123.22	154.87	923.66	474.73
	95% CI	-140.72/-31.37	192.61/451.41	-143.93/-34.30	-1.23/232.99	-38.74/194.15
Strategy SC	M	-86.66	238.95	-77.72	10.97	43.48*
	SD	67.27	142.08	88.11	208.04	142.25
	MIN	-233.82	50.79	-234.56	-553.95	-442.04
	**MED**	**-79.58** ^**A, a**^	**215.08** ^**A, a**^	**-82.15** ^**A, a**^	**-3.16** ^**A, a**^	**62.36** ^**A, a**^
	MAX	35.21	575.84	131.88	432.82	223.60
	95% CI	-114.43/-58.88	180.30/297.61	-114.09/-41.34	-74.90/96.84	-15.24/102.21

**Table 2:** Descriptive statistics of angle measurements with mean values (M), standard deviation (SD), median (MD) and 95% confidence interval (CI) of CEREC Primescan AC; uppercase superscript letters indicate significant differences between the scanning strategies regarding the trueness; lowercase superscript letters indicate significate differences between the scanning strategies regarding the precision

	Angular measurements overall (°)					Angular measurements coronal (°)					Angular measurements axial (°)				
Strategy	SD	M	MED	MIN/MAX	95% CI	SD	M	MED	MIN/MAX	95% CI	SD	M	MED	MIN/MAX	95% CI
FL	0.12	0.21	0.21^A, a^	0.00/0.57	0.15/0.27	0.13	0.17*	0.14^A, B, a^	0.00/0.56	0.11/0.23	0.06	0.09	0.08^A, a^	0.00/0.21	0.05/0.12
FZ	0.50	0.48*	0.36^B, a^	0.09/2.00	0.26/0.69	0.35	0.33`	0.25^B, a^	0.04/1.43	0.18/0.48	0.38	0.32‘	1.40^B, a^	0.17/1.4	0.15/0.48
FC	0.14	0.23*	0.22^A, a^	0.06/0.69	0.16/0.30	0.10	0.15	0.16^A, a^	0.00/0.31	0.11/0.20	0.15	0.14*	0.09^A, B, a^	0.00/0.68	0.07/0.20
HL	0.19	0.29*	0.26^A, a^	0.08/0.78	0.20/0.37	0.12	0.15	0.14^A, a^	0.01/0.4	0.10/0.21	0.18	0.22*	0.17^A, a^	0.05/0.72	0.14/0.30
HZ	0.21	0.31*	0.27^A, a^	0.08/1.01	0.21/0.40	0.14	0.18*	0.14^A, a^	0.01/0.47	0.11/0.24	0.21	0.22‘	0.17^A, a^	0.02/0.91	0.12/0.31
HC	0.17	0.29	0.27^A, a^	0.06/0.77	0.20/0.36	0.16	0.20	0.17^A, a^	0.00/0.65	0.13/0.27	0.13	0.17‘	0.12^A, a^	0.02/0.51	0.11/0.23
SL	0.32	0.32*	0.23^A, a^	0.05/1.59	0.17/0.45	0.15	0.21*	0.18^A, a^	0.03/0.65	0.14/0.27	0.32	0.19*	0.12^A, a^	0.01/1.58	0.04/0.33
SZ	0.36	0.46*	0.33^A, a^	0.07/1.32	0.17/0.66	0.19	0.30	0.32^A, a^	0.01/0.67	0.22/0.38	0.35	0.30‘	0.14^A, a^	0.01/1.22	0.15/0.45
SC	0.58	0.42*	0.25^A, a^	0.08/2.66	0.17/0.67	0.33	0.26*	0.21^A, a^	0.01/1.56	0.12/0.41	0.49	0.31‘	0.16^A, a^	0.04/2.16	0.10/0.52

Shapiro-Wilk test resulted in 1 out of 9 normally distributed group for the linear parameter, for the angular parameters 9 out of 9 were not normally distributed. For the trueness, statistically significant differences between the nine different strategies were found. The precision, determined on basis of the standard deviation, showed also differences between tested groups.

### Influence of movement pattern

For parameters V_E_ (*p* = 0.008), V_E_(y) (*p* = 0.023), $\:\alpha\:$_overall_ (*p* = 0.006) and $\:\alpha\:$_axial_ (*p* = 0.033), strategy F_L_ results in significantly higher trueness than strategy F_Z_. For V_E_ (*p* = 0.008), $\:\alpha\:$_overall_ (*p* = 0.006) and $\:\alpha\:$_coronal_ (*p* = 0.005) strategy F_C_ shows significantly better trueness than strategy F_Z_. Considering V_E_(z), strategy F_C_ resulted in better trueness than F_L_ and H_L_ than H_Z_ (*p* < 0.001 to *p* = 0.011).

For parameters V_E_ and V_E_(y) the angular parameters $\:\alpha\:$_overall_, $\:\alpha\:$_coronal_, $\:\alpha\:$_axial_, the significantly better trueness agrees with a better precision. Strategy F_L_ indicates a better precision compared to F_C_ for $\:{\stackrel{⃑}{\text{V}}}_{\text{E}}\left(\text{z}\right)$.

### Influence of scan segmentation

For parameters V_E_(y) (*p* = 0.005) and $\:\alpha\:$_axial_ (*p* = 0.002), strategy F_L_ showed significantly higher trueness than H_L_. For V_E_(z) (*p* = 0.001) strategy H_L_ resulted in significantly better trueness than F_L_ and S_L_.

Considering parameters V_E_(y) and $\:\alpha\:$_axial_, strategy F_L_ resulted in better precision than strategy H_L_. For parameter V_E_(z) strategy H_L_ showed better precision than strategy S_L_.

## Discussion

The present study evaluates the accuracy of CEREC Primescan AC depending on the scanning pattern used for in-vitro full-arch digitization. The impact of scanning strategy on the digitization accuracy has been demonstrated repeatedly in the literature [7, 21, 24]. To improve comparability, this study systematically combined three distinct motion patterns with three possible segmentations of the upper jaw. Compared to previously published literature, different strategies were defined and investigated. The null hypothesis of the present study, which stated that there would be no significant differences between the applied scanning strategies, has to be rejected, as significant differences could be observed.

For CEREC Primescan AC, two scanning patterns have been shown to be preferable to a third variation, with strategies involving linear movements proving to be advantageous. Consistent to the present results, Müller et al. [21] recommended the manufacturer’s specified scanning protocol, corresponding to strategy F_L_ in case of CEREC Primescan AC. Hence, it could be assumed that the constant movements would result in fewer interruptions during image acquisition [8]. Contrary, Passos et al. [8] reported improved accuracy using a strategy that combined linear and rotational movements.

In the present investigation, no significant differences have been observed between the scanning strategies regarding deviations along the X-axis. However, regarding the deviations in the Y- and Z-axes, strategies F_C_ and F_L_ exhibited a significantly better trueness than strategy F_Z_. Similar results were noted for the angular parameters α_overall_, α_coronal_, and α_axial_, which were also associated with improved precision. The lower trueness observed with zig-zag movements may be attributed to the absence of occlusal orientation structure, resulting in greater errors caused by inaccurate image overlap [21]. In addition, the varying motion patterns of zig-zag movements could be a disadvantageous due to the frequent change of the focal plane of the optical acquisition unit. This interpretation aligns with findings reported in the existing literature [8].

The trueness and precision for VE (y) and α_axial_ of strategy F_L_ were superior compared to strategy H_L_. Strategy F_L_ aligns with the manufacturer’s recommendation but involves the longest scan path distance in one turn. Thus, it can be assumed that a lower number of handpiece rotations may result in reduced interference during data acquisition [8]. Related to the Y-axis, it is conceivable that the scanning errors in the anterior-posterior direction accumulate due to the larger scan section. [19]. Supporting this presumption, Waldecker et al. [25] reported that linear deviations increase with the scanning path length. By dividing the jaw into minor sections of two or three segments, the authors of the present study aimed to minimize the accumulated error of the large scan segment within a complete dental arch. These findings can, with the present study, only be partially confirmed.

For the parameter V_E_(z), the strategy H_L_ demonstrated higher accuracy than the strategies F_L_ and S_L_. Similar findings were reported in a study using CEREC Omnicam (Dentsply Sirona, Bensheim, Germany) where strategy H_L_ also produced the best results in this context [22]. Nevertheless, as CEREC Omnicam and CEREC Primescan employ entirely different camera technologies, this comparison requires validation through testing with the same scanner model.

In the present study, accuracy was determined as trueness and precision according to ISO 5725-1 [26] and prior literature [13, 27]. According to this definition, trueness was assessed using the mean values of the linear and angular deviations between the test and reference data, while precision was estimated based on the standard deviation (SD). This was due to using actual one-dimensional measurements for a predefined geometric structure (the bar) rather than relying on best-fit alignments of the test dataset for the bar or the complete arch. Linear measurements were performed along the X-, Y-, and Z-axes, allowing for a more accurate assessment of the subsequent intraoral fit of the restoration. The successful in vivo application of this method has also been documented [13].

However, like every scientific work, the present work is subjected to several limitations.

It should be noted that this study investigates only the initial stage of the digital workflow. The accuracy of the fabricated prosthesis is influenced by several factors, including the number of interfaces, the CAD and the CAM of the restoration [28, 29]. For fixed prostheses, particularly those extending to a full arch, digitization plays a pivotal role by influencing linear and angular deviations, potentially resulting in restoration misfits. Unlike tooth-supported restorations, implant-supported restorations attached to osseointegrated implants lack the ability to compensate for these inaccuracies.

In this study, CEREC Primescan AC, a currently available model on the market, was used. The scanner employs a light optical measurement principle based on triangulation in combination with confocal principle to generate three-dimensional surface data [30]. As the dimension of the IOS handpiece may limit the application of the rotating/zig-zag scanning strategy in the molar region, a clinical trial is necessary for further investigation. Moreover, maintaining a complex scanning path such as “zig-zag” is more difficult in vivo than when scanning a model.

Since intraoral conditions - such as saliva, limited space, handling of soft tissues, light variations, and patient movements - are proven to affect the accuracy of IOS data [20, 24], further research focusing on full-arch scans under clinical conditions should be prioritized. Besides, for a more patient-like configuration, it would make sense to fix the reference object in the occlusal plane to be in the same focal plane and include more than one operator for the performance of the scans. CEREC Primescan AC offers an extraoral mode that was used for our in-vitro scans that could also be an influencing parameter on the generated data. According to Kuhr et al. [31], the scanning of the maxillary model results in the advantage of a larger surface area in the palatal region, which can be used as an orientation structure. Due to the anatomical shape of the model with physiologically shaped teeth, the study design is like a clinical setup. Therefore, future studies should incorporate adjusted scanning strategies in a clinical setup, include more scanning operators, and evaluate scanning devices from other manufacturers to provide comprehensive insights.

For CEREC Primescan AC the scanning pattern F_L_, F_C_, and H_L_ exhibited the best performance for trueness and precision. Overall, the findings of the current investigations suggest that linear and combined movements in combination with full jaw or half jaw segmentation (strategies F_L_, F_C_, and H_L_) are advantageous for CEREC Primescan AC regarding trueness and precision.

## Conclusions

Within the limitations of this study, it can be concluded that:

1. the combination of full arch with the linear or combined movement pattern (strategies F_L_ and F_C_) resulted in better trueness and precision for most measured parameters compared to the zig-zag movement (F_Z_).
2. the linear motion pattern in combination with full-arch or half-jaw segmentation (strategies F_L_, H_L_) showed significantly better trueness compared to sextant segmentation (S_L_) for linear measurement parameters.

Thus, scanning movement and scan segmentation have a significant influence in trueness and precision of full-arch scans. However, for clear recommendations of ideal scan paths for the clinical practice, further studies should be carried out.

## Data Availability

No datasets were generated or analysed during the current study.
