# Correction to: Accuracy of full arch scans performed with nine different scanning patterns– an in vitro study

**DOI:** 10.1007/s00784-025-06233-4

**Published:** 2025-02-18

**Authors:** Kerstin Schlögl, Jan-Frederik Güth, Tobias Graf, Christine Keul

**Affiliations:** 1 Department of Prosthetic Dentistry, LMU University Hospital, LMU Munich, Goethestrasse 70, 80336 Munich, Germany; 2 Department of Prosthetic Dentistry, Center for Dentistry and Oral Medicine (Carolinum), Goethe University Frankfurt am Main, Frankfurt am Main, Germany

**Correction to: Clinical Oral Investigations**

10.1007/s00784-025-06154-2

In the published paper, the name of the corresponding author was captured twice in the author group.

The original article has been corrected.

